# Feedback GAP: study protocol for a cluster-randomized trial of goal setting and action plans to increase the effectiveness of audit and feedback interventions in primary care

**DOI:** 10.1186/1748-5908-5-98

**Published:** 2010-12-17

**Authors:** Noah M Ivers, Karen Tu, Jill Francis, Jan Barnsley, Baiju Shah, Ross Upshur, Alex Kiss, Jeremy M Grimshaw, Merrick Zwarenstein

**Affiliations:** 1 Women's College Hospital Family Health Team, 76 Grenville Street, Toronto ON, M5 S 1B2, Canada; 2 Institute for Clinical Evaluative Sciences, 2075 Bayview Avenue, Toronto ON, M4N 3M5, Canada; 3 Department of Health Policy Management and Evaluation, University of Toronto, Health Sciences Building, 155 College Street, Suite 425, Toronto, ON, M5T 3M6, Canada; 4 Department of Family and Community Medicine, University of Toronto, 263 McCaul Street, 5th Floor Toronto ON, M5T 1W7, Canada; 5 Toronto Western Hospital Family Health Team, University Health Network, 399 Bathurst Street, West Wing, 2nd Floor, Toronto, ON, M5T 2S8, Canada; 6 Health Services Research Unit, University of Aberdeen, Third Floor Health Sciences Building, Foresterhill, Aberdeen, UK; 7 Sunnybrook Health Sciences Centre, 2075 Bayview Avenue, Toronto ON, M4N 3M5, Canada; 8 Joint Centre for Bioethics, University of Toronto, Health Sciences Building, 155 College Street, 7th floor, Toronto, ON, M5T 1P8, Canada; 9 Clinical Epidemiology Program, Ottawa Health Research Institute, 1053 Carling Avenue, Administration Building, Room 2-017, Ottawa ON, K1Y 4E9, Canada

## Abstract

**Background:**

Audit and feedback to physicians is commonly used alone or as part of multifaceted interventions. While it can play an important role in quality improvement, the optimal design of audit and feedback is unknown. This study explores how feedback can be improved to increase acceptability and usability in primary care. The trial seeks to determine whether a theory-informed worksheet appended to feedback reports can help family physicians improve quality of care for their patients with diabetes and/or ischemic heart disease.

**Methods:**

Two-arm cluster trial was conducted with participating primary care practices allocated using minimization to simple feedback or enhanced feedback group. The simple feedback group receives performance feedback reports every six months for two years regarding the proportion of their patients with diabetes and/or ischemic heart disease who are meeting quality targets. The enhanced feedback group receives these same reports as well as a theory-informed worksheet designed to facilitate goal setting and action plan development in response to the feedback reports. Participants are family physicians from across Ontario who use electronic medical records; data for rostered patients are used to produce the feedback reports and for analysis.

**Outcomes:**

The primary disease outcomes are the blood pressure (BP), and low-density lipoprotein cholesterol (LDL) levels. The primary process measure is a composite score indicating the number of recommended activities (*e.g*., tests and prescriptions) conducted by the family physicians for their patients with diabetes and/or ischemic heart disease within the appropriate timeframe. Secondary outcomes are the proportion of patients whose results meet targets for glucose, LDL, and BP as well as the percent of patients receiving relevant prescriptions. A qualitative process evaluation using semi-structured interviews will explore perceived barriers to behaviour change in response to feedback reports and preferences with regard to feedback design.

**Analysis:**

Intention-to-treat approach will be used to analyze the trial. Analysis will be performed on patient-level variables using generalized estimating equation models to adjust for covariates and account for the clustered nature of the data. The trial is powered to show small, but clinically important differences of 7 mmHG in systolic BP and 0.32 mmol/L in LDL.

**Trial Registration:**

ClinicalTrials.gov NCT00996645

## Background

Patients with diabetes or ischemic heart disease (IHD) are at elevated risk of cardiovascular events, especially if they have a history of both conditions [1]. Research findings regarding quality indicators in diabetes and IHD suggest agreement and acceptance of guidelines amongst Canadian family practitioners, who manage the bulk of care for these patients [2]. Unfortunately, there remains a large gap between ideal and actual care provided to such patients, making them a common focus for translational research [3]. Diabetes and IHD are considered particularly good targets for quality improvement strategies such as audit and feedback which can increase adoption and adherence to guidelines [4,5]. Audit and feedback has been defined as a 'summary of performance in a specific area with or without recommendations for action' [6] and is felt to be effective because it may overcome physicians' limited ability to accurately self-assess [7]. Thus, audit and feedback focuses on addressing the gap between ideal and actual care that is within the control of the health care provider and is often the foundation of multifaceted quality improvement interventions.

The Cochrane review of audit and feedback [8] concluded that it is effective, but the authors noted great variability in the design and the effectiveness of feedback interventions. That meta-analysis included 118 trials, finding a median increase in compliance with guidelines of 5% for dichotomous outcomes (inter-quartile range 3% to 11%) and 16% for continuous outcomes (inter-quartile range 5% to 37%), but great heterogeneity in both intervention design and reporting limited conclusions regarding how to best implement audit and feedback. Therefore, the important question to ask at this point is not whether feedback is effective, but how feedback can be most effective. Optimal design and delivery of feedback should more consistently lead to improved results, but few studies have tested different designs of feedback [9]. In an attempt to further delineate how to most effectively design and deliver feedback interventions, Hysong completed a re-analysis of the Cochrane review [10], finding that feedback had greater effectiveness with increasing frequency, with written rather than verbal or graphical delivery, and with feedback that included 'correct solution information.' Most recently, Gardner *et al*. conducted a reanalysis of the Cochrane review to test target-setting and action plans as effect-modifiers of feedback [11], but very few studies explicitly described their use of targets and/or action plans, making their report inconclusive.

### Using theory to design an enhanced feedback intervention

Briefly, performance feedback may be understood to work by directing attention toward a discrepancy between an expected or desired state and reality [12]. When present, this discrepancy may encourage the recipient to generate increased effort toward the goal, especially if the next steps or sub-goals are clear. Alternatively, the feedback may be perceived as inaccurate and disregarded, or the feedback may result in the recipient lowering their goal to make it more achievable [13]. To be effective, the ideally designed feedback would consistently lead to changes in behaviour by the recipient through increased efforts to reach appropriate goals.

There are both theoretical and empirical reasons to believe that feedback will be more effective if the recipients set goals [14] and develop action plans [15]. According to Goal-Setting Theory [16], those who are dissatisfied with their performance will develop a change in behaviour if they are committed to the goal and if they meet a threshold level of self-efficacy for that task. Bandura explains that people are more likely to try to accomplish a goal if they believe their efforts will be successful [17].

Psychologists have repeatedly shown that detailed plans regarding where/when/how behaviours will be enacted increases the likelihood of task accomplishment [18]. In the context of feedback and goals, these plans may increase goal-directed behaviours by increasing self-efficacy. Action plans can also facilitate success by increasing goal-commitment to overcome barriers such as distraction or fatigue; implementation plans in particular seem to increase goal-directed behaviours [19]. Implementation intentions are developed through if/then statements, wherein the participant must connect a situation (if) with a behavioural response (then). With some effort (*i.e*., considering and writing down the plan), the connection made between contextual cues and an action plan can become automatic, thereby increasing goal attainment without conscious intent.

**Figure 1:**
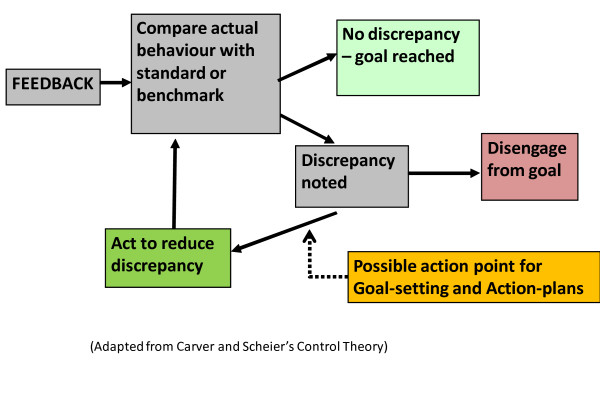
**Illustration of hypothesized role played by goal-setting and action-plan worksheet to promote increased effort to improve care**.

There is some empirical evidence that intensive interventions that help participants set practice improvement plans in response to feedback can improve outcomes [20], possibly by increasing goal commitment and self-efficacy. Recognizing that feedback alone is sometimes not enough to change provider behaviours, further improvements have been found by pairing feedback with more intensive (and expensive) co-interventions, such as educational outreach visits [21]. Although these intensive interventions are rarely explicitly informed by theory [22], they presumably work because they help participants to take action to improve outcomes for patients. The assumption is that healthcare providers intend to provide consistently high quality of care, but are uncertain how to change their behaviours to accomplish this feat. Therefore, an intervention aiming to close this intention-behaviour-gap could be very effective. In this study, it is expected that the feedback reports will draw family physicians' attention to a discrepancy between actual and ideal care -- *e.g*., fewer patients than expected are at target blood pressure (BP) -- and that a theoretically-informed worksheet can be developed to facilitate goal-setting and action-plans to enhance the likelihood that they will take action to close this gap (*e.g*., implement systems to monitor at-risk patients with BP above target). See Figure 1 for an illustration of potential role of goal-setting and action plans on effectiveness of feedback.

### Study objectives and hypotheses

For family physicians receiving performance feedback reporting the percentage of their patients with diabetes and/or IHD who are achieving quality targets, the addition of a theory-informed worksheet designed to facilitate goal setting and the development of action plans will lead to changes in behaviour and improved outcomes. A second hypothesis is that those physicians in the intervention group who properly complete the worksheet will have the largest improvement in outcomes. Finally, we aim to explore qualitatively the perceived barriers to behaviour change in response to feedback reports and the preferences of family physicians with regard to feedback design.

## Methods

### Study design

This is a mixed methods study built around a pragmatic, cluster-trial with two arms; one group will receive 'simple' feedback, while the other will receive 'enhanced' feedback. Allocation is at the practice level to reduce risk of contamination and the intervention directed at the physician level. In Ontario, family physicians do work in groups, but this generally involves sharing administrative resources, not patients. The usual approach is for chronic conditions to be dealt with by the personal physician, while acute issues may be dealt with by the available physician. Therefore, we believe that an intervention aimed at the physician rather than the entire clinic is appropriate. The analysis will be at the patient level, including both disease-level outcomes of BP and low-density lipoprotein cholesterol (LDL) and a composite score of process variables, as detailed in the 'outcomes' section.

The lack of a control group receiving no feedback at all is both necessary (because participants expected something in return for contributing data) and pragmatic (because most quality improvement interventions include some degree of feedback, making this the 'usual care' comparator) [23]. In particular, the Ministry of Health and Long-Term Care in Ontario (MOHLTC) is providing feedback reports to all family physicians as part of its diabetes strategy [24].

This study has received approval from the Research Ethics Office at Sunnybrook Health Sciences Centre (271-2006) and is registered with ClinicalTrials.gov (NCT00996645).

### Participants and data collection

Through the Electronic Medical Record Administrative data Linked Database (EMRALD), family physicians from all regions of Ontario who have been using Practice Solutions^® ^Electronic Medical Records (EMRs) for at least 12 months have signed a data-sharing agreement with the Institute for Clinical Evaluative Sciences (ICES) where EMRALD is held. ICES is a prescribed entity, under the province of Ontario's Personal Health Information Protection Act, which allows for the collection of individual level health information for use in planning and managing the healthcare system.

At ICES, mechanisms to extract, securely transfer, and de-identify the EMR data have been established[25]. For patient visits, consultations, investigations, and treatments, data in EMRALD compare well with (and often out-perform) administrative databases [unpublished data]. Furthermore, algorithms to identify patients in the EMRALD database with diabetes (sensitivity 83.1%, specificity 98.2%) [26] and IHD (sensitivity 72.4%, specificity 99.6%) [27] have been validated. Since publishing those papers, we have made further improvement to the diabetes algorithm (by considering the 'problem list' and 'past medical history' fields in addition to the lab tests and prescriptions) resulting in a sensitivity of 90.9% and specificity of 99.2%. The algorithm for identification of the IHD algorithm has also been improved by continuing to refine the search terms used to identify patients in the 'problem-list' and 'past medical history' fields. The algorithms developed for EMRALD do not require any special coding or data input by the participating physicians.

Family physicians were originally invited to participate in EMRALD through convenience sampling of EMR users. Presently, all participants work in multi-provider clinics and nearly all have access to allied healthcare providers. As a result, participants are not necessarily representative of all Ontario family physicians or of all primary care EMR users. However, this concern is partially mitigated by the varied characteristics of physicians in the sample (Table 1). As EMRALD continues to recruit new family physicians, the diversity of the sample is expected to grow. The number of participating physicians and practices in EMRALD may grow by as much as 50% during the first six months of this trial.

**Table 1:** Characteristics of initial 14 clinics and 54 family physicians in the trial

Clinic Characteristics (N = 14)				Physician Characteristics (N = 54)	
**Roster size, median (IQR)**				**Sex, n (%)**	

Total patients (total N = 46,864)	2,759 (1,915 to 4,250)			Male	30 (55.6%)

IHD or diabetes (total N = 4,593)	263 (219 to 505)				

				**Years in Practice, n (%)**	

**Location, n (%)**				Less than or equal to 10	17 (31.5%)

Rural	7 (50.0%)			11 to 20	12 (22.2%)

				More than 20	25 (46.3%)

**Physicians per clinic, n (%)**					

1 to 2	5 (35.7%)			**Years using EMR, n (%)**	

3 to 5	4 (28.6%)			Less than 3	5 (9.3%)

6 to 11	5 (35.7%)			3 to 6	45 (83.3%)

				More than 6	4 (7.4%)

N, n = number, IHD = ischemic heart disease, EMR = electronic medical record

For the present study, all EMRALD participants were sent consent forms and invitations to participate. Minimal eligibility criteria are applied. Physicians with less than one year of experience with EMR or with less than 100 rostered, active adult patients will be excluded, because these 'new' physicians would not have adequate data to assess some of the quality indicators. To be considered active, and to ensure there is a history with the physician, patients must have at least one visit between 12 and 36 months prior to the data upload date. The EMR data for all remaining physicians are assessed to ensure completeness with respect to electronic capture of lab tests and prescriptions. Finally, because ICES does not permit reporting of cells smaller than five (to ensure confidentiality), the data for the remaining physicians are assessed to ensure that each has more than five patients with diabetes and more than five with IHD.

### Setting

The Ontario Health Insurance Program pays for doctor visits and laboratory tests, but covers medications only for the elderly or those on social assistance. Over one-half of the primary care providers in Ontario have eschewed the old model of fee-for-service and joined primary care reform models where capitation plays a large role in compensation for patient care. To earn the capitation fees, physicians and patients must co-sign an agreement that officially adds the patient to the physician's roster; through this process, patients are encouraged to seek care primarily with their own provider or clinic. Only data from 'rostered' patients are included in the trial.

All the physician participants in this project roster their patients and most also benefit from the newest primary care reform process that provides funds to hire allied healthcare providers to work in the clinic. Although less than half of Ontario family physicians use EMR, Practice Solutions^® ^EMR has 45% of the Ontario EMR market [28].

### Intervention

The intervention has been developed through an iterative process. The materials for dissemination were designed after a review of the literature and consultation with experts from continuing medical education, health psychology, and knowledge translation.

Participants in both arms of the trial will each receive an information package by courier every six months for two years with multiple components, including a one-page cover letter, a one-page explanation of how the patient information was identified from EMRALD, a one-page handout reviewing generic clinical and quality improvement strategies for patients with diabetes and/or IHD (based in part on the chronic care model [29]), and two separate feedback reports. The first report will describe the percentage of the participating physician's patients with diabetes who are meeting evidence-based quality targets. The second will present similar information regarding their patients with IHD. The quality targets used were chosen to be consistent with those used by concurrent quality improvement interventions in Ontario (Quality Improvement and Innovation Partnership) [30] and with current guidelines (see Outcomes section below). The reports will present information comparing the performance achieved by the participating physician to the average achieved by the top 10% of participants for any given measure. This type of comparator is similar to the achievable benchmark of care previously shown to improve the effectiveness of feedback reports [31]. See Additional File 1 for prototype feedback reports.

Participants randomized to the enhanced feedback arm will receive exactly the same materials as the simple feedback arm, plus a one-page worksheet. This theory-informed worksheet is designed to facilitate participants in setting specific but challenging goals and help participants develop action-plans through the creation of implementation intentions (see Additional File 2 for prototype of worksheet). An evaluation to assess the theoretical validity of the intervention will be reported separately.

Based on our review of the literature, the largest effects from goal setting and action planning seem to come from actually developing the plan (and linking it to a specific context to carry it out). For this reason, we chose not to provide participants with a list of possible actions. The participants, not the investigators, decide how to improve upon a care gap that they identify as important. Important mediators of the success of implementation intentions seem to be participant adherence to instructions to develop an appropriate plan, participant self-efficacy, and the inclusion of 'coping plans' to help participants plan ahead for situations that could interrupt goal-oriented behaviours [32,33]. These factors will be addressed explicitly in this trial by: offering six 'Main-Pro-C' [34] continuing medical education credits to encourage full completion of the worksheet and to permit monitoring of plans by the investigators; allowing participants to set their own goals for improvement; and requiring participants to develop a coping plan in the intervention worksheet. The format in this aspect of the worksheet is similar to previous studies [33], although to our knowledge the application of this type of intervention to family physicians is novel.

The worksheet in this intervention is similar in concept to commitment-to-change procedures that are increasingly used in the continuing medical education field, based on multiple theories related to adult learning [35]. Rigorous evaluations of such procedures are few, but one study indicated that commitment-to-change can mediate the effect of an educational intervention for prescriptions [36]. Although a signature has not been proven to increase the effectiveness of the commitment-to-change procedure [37], it is included in the worksheet because it offers an opportunity to explicitly use the word 'commitment;' this is thought to be a necessary feature for the procedure to successfully generate behaviour change (see Additional File 2 for prototype of worksheet) [38]. We tested the worksheet design and all other intervention materials with a group of non-participating family physicians and they found it easy to use. Specifically, they reported that they found the instructions clear and advised no changes to the design. To our knowledge, the application of this type of worksheet as a means of 'enhancing' the effectiveness of audit and feedback is novel.

### Allocation and blinding

Given the small number of practices (clusters) involved in EMRALD, simple randomization cannot be expected to generate two similar arms for this trial. Instead, minimization was used to achieve balance at baseline across the three primary outcomes and the number of patients in each cluster who have diabetes and/or IHD. Using the baseline data for each cluster, these variables were classified as high or low using the median value as the cut-point. This was conducted with the free software, 'MINIM' [39]. Enrolment will continue for a maximum of six months; new practices will be minimized using their baseline data.

Although minimization has been criticised for increasing the risk for selection bias [40], the benefits of achieving balance at baseline through the use of minimization outweighs the minor risks involved (especially for small trials) and therefore has been widely recommended [41,42]. We believe that risk of selection bias is low in this case because the recruitment of the first fourteen practices (clusters) was completed prior to allocation of the practices using the minimization software. Furthermore, allocation of practices will remain concealed; study IDs will be used to ensure that the audit process and statistical analysis will be blinded with regard to group assignment. A research coordinator who is otherwise unaffiliated with the study will use a master list with study IDs and participant names to prepare the intervention materials. Participants will not be told explicitly which arm they are in; those in the enhanced feedback group will not be aware that the worksheet is the essential aspect of the intervention.

### Timeline

**Figure 2:**
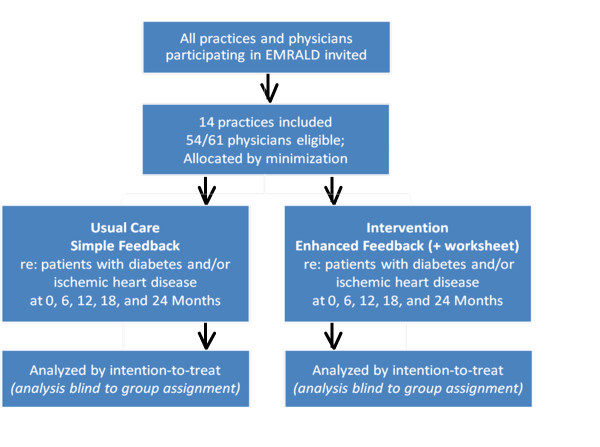
**Trial Design and Cluster-Patient Flow**.

The first reports were sent out in August 2010. Every six months, practices will send new data uploads to ICES so that new feedback reports can be produced and distributed. Final analysis will occur after an audit at 24 months. See Figure 2 for cluster-flow diagram.

### Analysis

#### Outcomes

There will be two disease primary outcomes and one process primary outcome. The disease primary outcomes will be the patients' most recent LDL and systolic BP values, if they have been tested within 24 or 12 months, respectively. Though these are actually risk factors, they are the target of cardiovascular risk management, and are therefore appropriate given the brevity of the trial.

The process primary outcome is a composite process score indicating whether patients with diabetes and/or IHD are receiving the recommended prescriptions and tests within the appropriate timeframes. These evidence-based quality indicators are concordant with guidelines [43-48] and are comparable to composite scores used in similar studies [47]. All patients with diabetes and/or IHD will receive a composite process score with a maximum of 6. For patients with diabetes, a maximum score of 6 would indicate testing of urinary microalbumin and serum LDL within a year, measuring BP and glycosylated haemoglobin within six months, and having active prescriptions for a statin and an angiotensin-modifying agent. For patients with IHD, a maximum score of 6 would indicate testing fasting blood glucose within two years and LDL within one year, measuring BP within six months, and having active prescriptions for aspirin, a statin, and an angiotensin-modifying agent. For patients with both diabetes and IHD, the maximum raw score will be 7 (based on the same indicators as diabetes but adding aspirin), but this will then be multiplied by 6/7 to standardize to a maximum score of 6, as outlined in Table 2.

**Table 2:** Composite process score to be calculated for each patient as primary process outcome; the score is calculated differently for patients with diabetes, ischemic heart disease, or both.

Quality indicator (for each patient receives a score)	Diabetes (max score = 6)	IHD (max score = 6)	Both Diabetes + IHD (multiply by 6/7 for max score = 6)
BP test in 6M	X	X	X

A1C test in 6M	X		X

FBG test in 24M		X	

LDL test in 12M	X	X	X

ACR test in 12M	X		X

Rx ASA		X	X

Rx Statin	X	X	X

Rx ACE/ARB	X	X	X

IHD = ischemic heart disease, BP = blood pressure, A1C = glycolated haemoglobin, FBG = fasting blood glucose, LDL = low density lipoprotein cholesterol, ACR = urinary albumin-to-creatinine ratio, ASA = aspirin, ACE/ARB = angiotensin-modifying agent

Secondary outcomes were chosen because they may be the targets of action by recipients of the intervention. Thus, the secondary outcomes include: the proportion of patients whose results meet the targets recommended in guidelines for glucose, LDL, and BP; and the prescriptions rates for insulin, beta blockers, angiotensin-modifying agents, aspirin, and statins.

## Analysis

Descriptive statistics will be calculated for all variables of interest. Continuous measures will be summarized using means and standard deviations whereas categorical measures will be summarized using counts and percentages.

We hypothesize that the enhanced feedback intervention will lead to greater improvements in quality of care for patients with diabetes and/or IHD. The analysis to test this hypothesis will be carried out using multilevel hierarchical modeling (using the generalized estimating equation approach) to control for the effects of clustering as well as adjusting for multiple covariates, including the variables used in the minimization (baseline values for BP, LDL, the composite process score, and the number of patients with diabetes and/or IHD). Analysis will be performed on an intention-to-treat basis. No interim analyses are planned. Prior to analysis, other covariates will be assessed for the presence of multicollinearity; when the tolerance statistic value < 0.4 only one member of a correlated set will be retained for the model. Primary analyses will be conducted on patient level variables, combining patients with diabetes and/or IHD. Sub-group analyses will be performed on patients with only IHD, only diabetes, or both, to assess the same outcome variables.

The efficacy of the worksheet intervention will be assessed as a planned secondary analysis in two ways. First, we will test whether full completion of the worksheet resulted in improved outcomes. Full completion of the worksheet will be evaluated according to whether they declared specific and measurable goals, completed all sections of the action plan, and confirmed their commitment with their signature. Second, we will examine if physicians achieved greater improvements in the specific clinical topics that they chose to target using the worksheet.

All analyses will be carried out using the SAS Version 9.2 statistical program (SAS Institute, Cary, NC, USA).

### Sample size

Based on pilot data, systolic BP is expected have a standard deviation of 20 mmHg. A clinically important difference in systolic BP is estimated to be 7 mmHg; this is a difference often seen with initiation of treatment and is associated with reduction in cardiovascular risk [49]. To have 80% power to find a difference in systolic BP of 7 mmHg using a two-sided unpaired t-test with α = 0.05 would require 258 total patients. To account for clustering, this sample size must be multiplied by a variance inflation factor (VIF) = [1 + (n - 1) × ICC], where n is the mean cluster sample size and ICC is the intra-class correlation coefficient, a measure of the degree of correlation within clusters [50]. From baseline data, the mean cluster size (number of patients with diabetes and/or IHD in each practice) is approximately 328. Using a presumed ICC of 0.05 (based on ICCs seen in the literature[51]), the VIF equals 17.4. Thus, 4,489 patients with diabetes and/or IHD are required to find a difference of 7 mmHg in BP, which equates to 13.7 clusters.

For LDL values, pilot data show a standard deviation of 0.90. Therefore, using the same calculations, the trial will have power to show an absolute difference in LDL of 0.32 mmol/L; this difference has been shown to be associated with reduction in cardiovascular risk [52]. This type of small improvement in the management of these very common chronic diseases could translate into a large impact on the population scale.

Based on pilot data, the standard deviation for the composite process primary outcome is expected to be 1.61. For this outcome, pilot data were also used to find that the ICC was 0.0059, but to be conservative this can be rounded up to 0.01, giving a VIF of 4.28. Therefore, to show an absolute difference in the final composite process score of 0.3 (effect size 0.19), a sample size of 3,878 patients would be needed, which equates to 11.82 clusters.

Most of the power in cluster-trials comes from the number of clusters, rather than the number of patients. Therefore, dropout of a few participating physicians (or many of their patients) would only minimally decrease power. We do not expect dropout of entire clinics; clinic managers are committed to this project and have facilitated the recruitment of individual physicians at each clinic. However, even with a loss of two of the fourteen clinics, the same calculations indicate that we would have 80% power to find differences of 8 mmHG BP or 0.36 mmol/L LDL.

### Qualitative analysis and process evaluation

Previous qualitative studies have isolated timeliness, customizability, and a non-punitive tone as key criteria for 'actionable' feedback [53]. Evidence from the organizational literature suggests that the recipient must be satisfied with the feedback for it to be accepted and acted upon [54]. Unfortunately, the literature has not provided clear direction regarding how to design feedback interventions targeted at family physicians to accomplish this goal.

One previous study has assessed eight Ontarian physicians' reactions to a 20-minute one-on-one performance assessment presentation based on chart audits and patient questionnaires, and found that physicians welcomed it [55]. Even though the data were garnered directly from charts, the participants expressed concerns about government involvement in the performance improvement process. Another study revealed a general scepticism amongst physicians regarding quality improvement interventions based on secondary databases [56]. Nevertheless, the MOHLTC, is now using administrative data to send all family physicians 'Diabetes Testing Reports' regarding their patients with diabetes [57]. These reports from the government will provide far less data to the physicians (and nothing regarding patients with IHD) compared to the intervention described in this protocol. This context provides an opportunity to work with physicians receiving two types of diabetes feedback to explore the barriers and facilitators to Ontario family physicians' acceptance and utilization of performance feedback, and to examine the perceived actionability of various approaches to the design and delivery of feedback. While the ongoing government feedback will likely enrich the qualitative component of the study, we do not believe that it will impact the inferences made from the trial. All participants will receive the government feedback, but the government feedback does not explicitly encourage goal setting or action plans.

Semi-structured, individual interviews will be conducted using an interview guide, developed based on a review of the literature and consideration of the twelve domains described by Michie *et al*. to explain behaviour change in response to an intervention [58]. We will use 'stratified purposeful sampling' [59]; we will select participants with those features believed to be relevant, not with the goal of probabilistic representativeness, but for informational representativeness. For instance, guideline adherence and quality of care may be inversely related to years in practice [60] and physician gender [61], so variety will be sought in these factors. Additionally, the participants will be chosen to represent varying levels of baseline performance, because this was found to be an important predictor in the Cochrane review. It is expected that saturation may be accomplished with approximately 12 interviews [62]. The sample will be weighted with about two-thirds of participants having received the enhanced feedback. To account for time away from patient care, an honorarium will be offered for participation.

Interviews will be recorded and a transcription service used to produce verbatim electronic transcripts. These will be stored with encryption software on a password-protected computer drive. Identifying factors will be omitted. NVivo™ software will be used to assist with the data analysis. The framework analysis approach, as described Ritchie and Spencer [63] (and more succinctly by Pope *et al*.[64]) aims to accurately reflect the original accounts of the participants through the use of inductive techniques, yet start out deductively with preset goals. As such, it represents an ideal foundation for analyzing qualitative data within a pragmatic, mixed methods study such as this one. For example, it has been successfully used in the past as part of a mixed method study investigating barriers and facilitators to guideline uptake in the ICU [65].

The identification of themes will be tracked along with dates of interpretations to provide an audit trail documenting the analysis. This is one way that trustworthiness in the results can be increased [66]. Next, an index of themes will be developed by combining *a priori *objectives and issues identified in the literature with those raised by the participants and recognized through the readings. This process will occur after seven interviews have been completed and will be repeated in part by a second researcher (JB). It is thought that multiple coding provides a system of check and balances to ensure that all possible themes are given consideration [67]. Disagreements will be settled through consensus and this process may lead to changes in the interview guide. At this point, disconfirming evidence to ensure saturation of themes will be sought from further participants through the use of snowball sampling (by asking participants to suggest colleagues that may have unique perspectives on feedback). In this way, elements of multiple coding and the constant comparative method will be incorporated. Therefore, the qualitative protocol will meet the criteria described by Kuper for judging qualitative research [68].

## Discussion

With the use of audit and feedback interventions likely to increase over time, there is a need to understand how their design effects the behaviour of primary care providers. This project will play a role in learning how to generate feedback reports that are useful for family physicians. Even if the trial is negative, the qualitative process evaluation will provide useful information and generate new study questions. Future studies may then compare the cost-effectiveness of using interventions similar to the one described in this trial against more intensive interventions such as academic detailing or practice facilitators.

There are also some important limitations that warrant discussion. One concern regarding generalizability is that it is possible that there will be a specific interaction between the worksheet intervention and the feedback such that adding the worksheet to other designs/types of feedback or other interventions may not work as well. It is also important to note that participants in EMRALD are a convenience sample of Ontario family physicians using Practice Solutions^®^. Therefore, findings from this group will not be strictly generalizable to other providers who use different EMR systems, or to those not using EMR at all. In addition, many of the clinics are involved in other quality improvement interventions. Thus, these clinics may be more innovative and may also be achieving a higher level of evidence-based care than most other primary care providers.

Perceived data accuracy has been identified as an essential feature for acceptance of feedback reports [69], and previous studies have shown that data quality from EMRs are uncertain [70]. While we have validated algorithms for identifying patients who have diabetes and/or IHD in this database, the automated chart audits that are the basis for both the intervention and the outcome assessment may occasionally have errors. However, there is no reason to believe that any such errors occurring in the data abstraction process represent a risk for bias; any problems with the algorithm would be equally likely to occur in either arm of the trial.

This is a pragmatic trial that is powered to find small (but important) effects of a feedback intervention based on the addition of a theoretically-informed worksheet. While a longer, larger trial would be ideal to assess true cardiovascular endpoints, the size and timeframe of this trial have been designed to ensure feasibility. Psychological theory applicable to feedback interventions indicates that the worksheet intervention tested in this trial should help family physicians to close the gap between their intended and actual behaviours with respect to the care they provide for their patients. If the intervention works in this sample, it could be tested more broadly; if it is not effective, it may be necessary to try more intensive approaches to facilitate providers to achieve their quality improvement targets.

## Competing interests

The authors declare that they have no competing interests.

## Authors' contributions

NI and MZ conceived the idea. NI prepared the manuscript. All authors have made substantial contributions to the research design, have edited the manuscript critically, and have approved of the final version.

## Supplementary Material

Additional file 1 **Feedback Intervention**. Prototype of feedback report that all participants will receive Click here for file

Additional file 2 **Goal-setting and Action-plan Worksheet for Enhanced Feedback Intervention**. Prototype of the intervention that will be tested in the trial Click here for file
