# Supplementary material for: Feedback GAP: study protocol for a cluster-randomized trial of goal setting and action plans to increase the effectiveness of audit and feedback interventions in primary care
Source: Implement Sci. 2010 Dec 17;5:98. doi: 10.1186/1748-5908-5-98 (PMC3161381; doi:10.1186/1748-5908-5-98)
Supplement: Additional file 2 — Goal-setting and Action-plan Worksheet for Enhanced Feedback Intervention. Prototype of the intervention that will be tested in the trial [file 1748-5908-5-98-S2.DOCX]

**1. Describe a goal that you will achieve *within the next 6 months* for your diabetic patients and for your IHD patients. Your goal must be challenging but achievable. Be very specific.**

[Phrase your goal as follows: “I will improve *(choose one of the outcomes in the practice profile e.g. % at target BP)*

to the goal of *(state a target for your efforts e.g. by 20 percentage points)”*]

For Diabetes, I will improve:

To the goal of:

For Ischemic heart disease (IHD), I will improve:

To the goal of:

**2. Complete the following statements by describing a specific action you will take to help you achieve your goal:**

*To identify on an ongoing basis the specific patients in my practice who are not meeting targets, I will:*

*If a patient with Diabetes and/or IHD comes to clinic (for any reason) and is not meeting targets, I will:*

*If I am too busy during an office visit to address all aspects of managing the patient’s diabetes and/or IHD, I will:*

*If I’m not making progress with respect to implementing my plan for achieving my goals, I will:*

In signing below, I confirm my commitment to achieve this goal and my intention to carry out this action plan.

_________________________________ __________________

Signature Date
